# Diagnostic Yield and Treatment Impact of Targeted Exome Sequencing in Early-Onset Epilepsy

**DOI:** 10.3389/fneur.2019.00434

**Published:** 2019-05-21

**Authors:** Michelle Demos, Ilaria Guella, Conrado DeGuzman, Marna B. McKenzie, Sarah E. Buerki, Daniel M. Evans, Eric B. Toyota, Cyrus Boelman, Linda L. Huh, Anita Datta, Aspasia Michoulas, Kathryn Selby, Bruce H. Bjornson, Gabriella Horvath, Elena Lopez-Rangel, Clara D. M. van Karnebeek, Ramona Salvarinova, Erin Slade, Patrice Eydoux, Shelin Adam, Margot I. Van Allen, Tanya N. Nelson, Corneliu Bolbocean, Mary B. Connolly, Matthew J. Farrer

**Affiliations:** ^1^Division of Neurology, Department of Pediatrics, University of British Columbia and BC Children's Hospital, Vancouver, BC, Canada; ^2^Department of Medical Genetics, Centre for Applied Neurogenetics (CAN), University of British Columbia, Vancouver, BC, Canada; ^3^Division of Neuropediatrics, University Children's Hospital Zurich, Zurich, Switzerland; ^4^Division of Biochemical Diseases, Department of Pediatrics, BC Children's Hospital, University of British Columbia, Vancouver, BC, Canada; ^5^Division of Developmental Pediatrics, Department of Pediatrics, BC Children's Hospital, University of British Columbia, Vancouver, BC, Canada; ^6^Department of Pediatrics, Centre for Molecular Medicine and Therapeutics, BCCHRI, University of British Columbia, Vancouver, BC, Canada; ^7^Department of Pediatrics, Academic Medical Centre, Amsterdam, Netherlands; ^8^Division of Genome Diagnostics, Department of Pathology and Laboratory Medicine, BC Children's Hospital, Vancouver, BC, Canada; ^9^Department of Pathology and Laboratory Medicine, University of British Columbia, Vancouver, BC, Canada; ^10^Department of Medical Genetics, BC Children's and BC's Women's Hospitals, University of British Columbia, Vancouver, BC, Canada; ^11^University of Tennessee Health Science Center, Memphis, TN, United States; ^12^Centre for Addiction and Mental Health, Toronto, ON, Canada

**Keywords:** targeted WES, early-onset epilepsy, diagnostic yield, cost estimation, Canada

## Abstract

Targeted whole-exome sequencing (WES) is a powerful diagnostic tool for a broad spectrum of heterogeneous neurological disorders. Here, we aim to examine the impact on diagnosis, treatment and cost with early use of targeted WES in early-onset epilepsy. WES was performed on 180 patients with early-onset epilepsy (≤5 years) of unknown cause. Patients were classified as Retrospective (epilepsy diagnosis >6 months) or Prospective (epilepsy diagnosis <6 months). WES was performed on an Ion Proton™ and variant reporting was restricted to the sequences of 620 known epilepsy genes. Diagnostic yield and time to diagnosis were calculated. An analysis of cost and impact on treatment was also performed. A molecular diagnoses (pathogenic/likely pathogenic variants) was achieved in 59/180 patients (33%). Clinical management changed following WES findings in 23 of 59 diagnosed patients (39%) or 13% of all patients. A possible diagnosis was identified in 21 additional patients (12%) for whom supporting evidence is pending. Time from epilepsy onset to a genetic diagnosis was faster when WES was performed early in the diagnostic process (mean: 145 days Prospective vs. 2,882 days Retrospective). Costs of prior negative tests averaged $8,344 per patient in the Retrospective group, suggesting savings of $5,110 per patient using WES. These results highlight the diagnostic yield, clinical utility and potential cost-effectiveness of using targeted WES early in the diagnostic workup of patients with unexplained early-onset epilepsy. The costs and clinical benefits are likely to continue to improve. Advances in precision medicine and further studies regarding impact on long-term clinical outcome will be important.

## Introduction

Epilepsy is a common pediatric neurological disorder associated with an increased risk of developmental delay, autism and psychiatric illness; and for which treatment is ineffective in 30–40% of patients. High-throughput sequencing (HTS) has become a widespread diagnostic tool in various genetic conditions, including epilepsy (1), vastly improving molecular diagnosis. Its clinical utility has been proven in epileptic encephalopathies and in mixed epilepsy cohorts (2–11); and in neurodevelopmental disorders (12–14) in which epilepsy is a comorbid feature. The diagnostic yield ranges between 10 and 60%, settling around 25% in large studies with broader inclusion criteria, and with comparable diagnostic rates between gene panels and WES (1). A genetic diagnosis of epilepsy may enable more accurate counseling regarding prognosis and recurrence risk, avoids unnecessary medical investigations and may change care. It also allows families to connect with the same genetic condition and/or join support groups. Recent studies have demonstrated the potential cost savings of WES in the diagnostic work-up of children with suspected monogenic disorders (15–22). However, in Canada, access to such technology in clinical care is variable. In this British Columbia study, we assess the effectiveness of using WES by comparing diagnostic yield, time to diagnosis, and cost to current clinical practices. The potential treatment impact of a genetic diagnosis is also described.

## Methods

### Patients

One-hundred and eighty patients with epilepsy (23) were enrolled between December 2014 and September 2018. All had seizure onset at ≤5 years of undefined cause after clinical evaluation, EEG, brain MRI and chromosome microarray investigations. Seizure types and electroclinical syndromes were classified according to the International League Against Epilepsy (ILAE) (24). Patients with self-limiting benign electroclinical syndromes, such as Childhood Absence Epilepsy (onset >4 years), were excluded as they most likely have multifactorial inheritance. Patients were classified as **Retrospective** (*n* = 127), defined as an epilepsy diagnosis >6 months before study enrollment with a standard clinical approach to genetic testing (variable genetic tests which include gene-by-gene approach using Sanger sequencing, small epilepsy gene panels using high-throughput sequencing, and/or mitochondrial DNA sequencing; or **Prospective** (*n* = 53), which included an epilepsy diagnosis <6 months before study enrollment date and having limited to no genetic testing. Varying degrees of screening tests for inborn errors of metabolism; such as plasma amino acids, lactate and ammonia, were also performed in both groups. Clinical data was recorded using a secure Research Electronic Data Capture (REDCap) (25) information system hosted at the Child and Family Research Institute.

This study was approved by the BC Children's Hospital and University of British Columbia Ethics Board (protocol number H14-01531). Informed consent and/or assent were obtained before study inclusion in accordance with the Declaration of Helsinki.

### Whole-Exome Sequencing

Genomic DNA was extracted from peripheral blood lymphocytes following standard protocols. Exonic regions were captured using the Ion AmpliSeq Exome Kit (57.7 Mb) and WES was performed on an Ion Proton™ according to manufacturers' recommendations (Life Technologies Inc., CA) within 2 weeks of receiving samples. Reads were aligned against the human reference genome hg19. Variant annotation was performed with ANNOVAR (26) integrating data from PHAST PhyloP (27), SIFT (28), Polyphen2 (29), LRT (30), and MutationTaster (31) algorithms, Combined Annotation Dependent Depletion (CADD) scores (32), dbSNP (www.ncbi.nlm.nih.gov/SNP/), the Genome Aggregation Database (gnomAD; gnomad.broadinstitute.org) and ClinVar (33) (www.ncbi.nlm.nih.gov/clinvar). Additionally, variants were compared to an in-house database containing more than 900 exomes to exclude platform artifacts and common variants not present in public databases.

Analysis was restricted to 620 genes previously implicated in epilepsy (Supplementary Table 1), using a gene-reporting pipeline developed in-house. The gene list was compiled through the combination of a comprehensive literature search (Pubmed, OMIM) and clinically available epilepsy panels. Annotation was limited to exonic non-synonymous and splicing (±5 bp) substitutions. Homozygous variants, potential compound heterozygous variants (defined as genes with >1 variant locus per individual) with a minor allele frequency (MAF) <1% and heterozygous variants with MAF <0.01% were reported. All samples were required to meet minimum quality standards, with a WES average coverage >80X.

Sanger sequencing, performed as previously described (34), was used on a case-specific basis in a few individuals with very specific clinical phenotypes to complete regions of poor coverage in genes related to the patient's phenotype when no candidate variants were identified, or when a heterozygous and potentially pathogenic variant was identified in gene previously implicated in autosomal recessive disease. No additional variants were identified though post-WES Sanger sequencing.

### Variant Prioritization and Validation

Cases were reviewed at a bi-weekly meeting by a multi-disciplinary genomic team. Variant prioritization was performed based on: (1) frequency in public databases; (2) predicted protein impact; (3) disease inheritance, and; (4) correlation of patient phenotype and candidate gene literature. Up to 3 putative causative variants were validated by Sanger sequencing in patient and parental samples. Clinical Sanger sequencing confirmation and interpretation in accordance with ACMG guidelines (35) allowed disclosure to families and management adjustments when indicated. Two time intervals were measured for the first 50 patients: (1) from a clinical diagnosis of epilepsy to Sanger validation of a putative pathogenic variant; and (2) from enrollment with genetic counseling to Sanger validation of the same.

### Additional Analysis

Exome data was periodically reanalyzed in patients, if no genetic diagnosis was identified in the initial analysis. Moreover, trio WES was performed in 27 patients (22 Retrospective and 5 Prospective) with no diagnosis.

### Genetic Counseling and Treatment Implications

Pre- and post-test genetic counseling was performed for each patient/family. As only a limited set of 620 genes related to seizure disorders were annotated, and only in affected probands, reporting related to incidental (secondary) findings was uncommon (36). Genetic disorders with specific therapeutic implications (47 genes) were defined as conditions in which current literature supports a preferred antiepileptic medication and/or approach (37–39).

### Cost Estimation

For the first 50 patients, resource use data were retrospectively acquired from electronic health records and medical charts. Cost estimates in Canadian dollars were based on micro-cost information from the British Columbia Provincial Medical Service Plan Index (2015), Canadian Interprovincial Reciprocal Billing Rates (2014/2015), Children's and Women's Health Center of British Columbia Internal Fee Schedule (2015) and the internal accounting system. Diagnostic costs included: biochemical tests, imaging tests, genetic tests, neurophysiological tests, and biopsies (a complete list of tests is provided in Figure Appendix 1). Academic and/or hospital pricing is used throughout. Inpatient hospitalization costs, outpatient visits such as clinic visits, and indirect costs such as parental time off work for medical visits related to their child's epilepsy were not included. All categorical and quantitative variables were analyzed using STATA (Release 13, College Station, TX).

## Results

Targeted WES was performed on 180 subjects and clinical features are summarized (Supplementary Table 2-first 50 patients are indicated in bold). Detailed clinical information has previously been reported for subjects 002, 013, 044, 073, and 144 (40–43). The average age of epilepsy onset was 18 months (range 0.03–60 months), 16 months for Prospective cases (*n* = 53) and 18 months for Retrospective cases (*n* = 127) (Tables 1, 2). Of the 620 genes, 87% had at least 80% of their consensus-coding region sequenced with >20X coverage (Supplementary Table 1).

**Table 1:** Results: Demographics and diagnostic yield.

	**All Patients (*N* = 180)**	**Prospective (*N* = 53)**	**Retrospective (*N* = 127)**
Age at Epilepsy Onset (months) average (range)	18 (0.03–60)	16 (0.1–60)	18 (0.03–60)
Males; Females	77;103	27;26	50;77
**DIAGNOSIS**			
Definite/likely	59 (33%)	21 (40%)	38 (30%)
**Treatment Implications**	**27 (46%)**	**15 (71%)**	**12 (32%)**
Possible	21 (12%)	4 (8%)	17 (13%)

*Patients highlighted in bold are those for whom a genetic diagnosis had treatment implications*.

**Table 2:** Patients with definite/likely diagnosis and treatment impact.

**ID Sex**	**AAO**	**Epilepsy type**	**Gene (IP)**	**GRCh37/hg19**	**Variant**	**Zyg**	**Origin**	**Treatment Impact**
**PROSPECTIVE PATIENTS**								
**001 M**	3 m	Dravet	*SCN1A (AD)*	chr2:166848491	NM_001165963:c.5294T>C;p.F1765S	het	*De novo*	Change from levetiracetam to clobazam, valproic acid, and topiramate
010 F	18.3 m	Unclassified	*SMC1A (XLD)*	chrX:53409269	NM_006306:c.3321C>G;p.Y1107X	het	*De novo*	
018 F	6 m	Ohtahara, West	*STXBP1 (AD)*	chr9:130434396	NM_003165:IVS12+1GT>AA;NA^c^	het	*De novo*	
**033 F**	9.2 m	EE	*POLG (AR)*	chr15:89871929	NM_002693:c.1157G>C;p.R386P	comp het	♀carrier	Stopped valproic acid; early palliative care; prenatal testing for next pregnancy
				chr15:89866657	NM_002693:c.2243G>C;p.W748S		♂carrier	
**059 M**	24 m	Unclassified	*GABRA1 (AD)*	chr5:161322788	NM_000806:c.973T>C;p.F325L	het	*De novo*	
069 M	51.5 m	Unclassified	*MED23 (AR)*	chr6:131944505	NM_015979:c.382G>A;p.G128R	comp het	♀carrier	
				chr6:131941826	NM_015979:c.539C>A;p.A180D		♂carrier	
**098 M**	1.5 m	Unclassified	*KCNT1 (AD)*	chr9:138660693	NM_020822:c.1420C>G;p.R474G	het	*De novo*	No change in management
**104 M**	0.9 m	SLFNE	*KCNQ2 (AD)*	chr20:62059782	NM_172107:c.154delT;p.I385TfsTer4	het	♀carrier	Stopped phenobarbital at 2 m; avoided MRI with anesthetic; seizure free and normal development at 6 m
**120 F**	1.8 m	West syndrome	*ADSL (AR)*	chr22:40754948	NM_000026:c.563G>A;p.R188H	comp het	♀carrier	S-Adenosyl-I-methionine trial proposed but patient died just prior to implementation
				chr22:40760969	NM_000026:c.1277G>A;p.R426H		♂carrier	
144 F	3 days	Unclassified	*FGF12^*a*^ (AD)*	chr3:192053223	NM_021032:c.341G>A;p.R114H	het	*De novo*	Remains seizure free with normal development on carbamazepine
**165 M**	21.6 m	Dravet	*SCN1A (AD)*	chr2:166915162	NM_001165963:c.301C>T;p.R101W	het	*De novo*	Avoiding sodium channel blockers and started Valproic acid early
**167 F**	7.7 m	Unclassified	*SCN8A (AD)*	chr12:52200900	NM_014191:c.5630A>G;p.N1877S	het	♂carrier	Kept on Sodium channel blockers. Seizure free and normal development
193 M	1.5 m	West syndrome	*ARX (XLR)*	chrX:25022869	NM_139058:c.1607G>C;p.R536T	hemi	♀carrier	
**197 M**	4.4 m	Dravet	*SCN1A (AD)*	chr2:166866246	NM_001165963:c.3985C>T;p.R1329X	het	*De novo*	Avoiding sodium channel blockers and started Valproic acid early
**200 F**	8.9 m	Dravet	*SCN1A (AD)*	chr2:166859067	NM_001165963:c.4198delA;p.I1400X	het	Unknown	Avoiding sodium channel blockers and started Valproic acid early
**201 M**	4.6 m	Dravet	*SCN1A (AD)*	chr2:166848872	NM_001165963:c.4913T>A;p.I1638N	het	*De novo*	Avoiding sodium channel blockers and started Valproic acid early
**205 M**	3 days	EE	*SCN1A (AD)*	chr2:166848429	NM_001165963:c.5356C>G;p.L1786V	het	*De novo*	Influenced medication choice: topiramate
**206 M**	4.6 m	Unclassified	*PRRT2 (AD)*	chr16:29824973	NM_145239:c.604_607delTCAC;p.S202HfsTer26	het	♂carrier	No change in management
222 F	52.1 m	EE	*MECP2 (XLD)*	chrX:153296878	NM_004992:c.401C>G;p.S134C	het	*De novo*	
**234 M**	0.8 w	Unclassified	*ALDH7A1 (AR)*	chr5:125882034	NM_001182:c.1547A>G;p.Y516C	het	♀carrier	Treated with B6, lysine restricted diet
				chr5:125887751	NM_001182:c.1279G>C;p.E427Q	het	♂carrier	
**237 F**	1 m	SLFIE	*SCN2A (AD)*	chr2:166231307	NM_021007:c.4085A>T;p.K1362M	het	♀carrier	Discontinue medication early because of predicted benign course
**RETROSPECTIVE PATIENTS**								
**002 M**	3.2 m	Dravet-like	*ATP1A2 (AR)*	chr1:160100072	NM_000702:c.1642C>T;p.R548C	comp het	♀carrier	Stopped stiripentol; started flunarizine. No further episodes on flunarizin
				chr1:160109762	NM_000702:c.3022C>T;p.R1008W		♂carrier	
005 F	7 m	West syndrome	*ALG13 (XLD)*	chrX:110928268	NM_001099922:c.320A>G;p.N107S	het	*De novo*	
006 M	10.4 m	LGS	*GABRB3 (AD)*	chr15:27017551	NM_000814:c.238A>G;p.M80V	het	Not ♀, ♂ NA	
013 F	5 m	EE	*KCNQ5^*a*^ (AD)*	chr6:73821107	NM_001160133:c.1106C>G;p.P369R	het	*De novo*	
014 M	0.5 w	EE	*PNPT1^*b*^ (AR)*	chr2:55910954	NM_033109:c.419C>T;p.P140L	hom	♀ & ♂ carrier	
023 M	11.2 m	EE	*PIGA (XLR)*	chrX:15344061	NM_002641:c.823C>T;p.R275T	hemi	♀carrier	
**039 M**	1 day	EE	*KCNQ2 (AD)*	chr20:62070997	NM_172107:c.881C>T;p.A294V	het	*De novo*	Topiramate changed to carbamazepine: no improvement in seizure frequency 6 months later
**040 F**	3 days	EE	*KCNQ2 (AD)*	chr20:62071034	NM_172107:c.844G>T;p.D282Y	het	*De novo*	Phenytoin changed to carbamazepine: seizures less frequent and shorter 9 months later
043 F	3 m	West syndrome	*PAFAH1B1 (AD)*	chr17:2577530	NM_000430:c.849_853delCTGGG;p.W292SfsTer10	het	*De novo*	
044 M	2.1 m	EE	*SLC1A2^*b*^ (AD)*	chr11:35336636	NM_004171:c.244G>A;p.G82R	het	*De novo*	
050 M	13.7 m	Unclassified	*TUBB2B (AD)*	chr6:3226887	NM_178012:c.74G>A;p.S25N	het	*De novo*	
063 M	18.5 m	Unclassified	*RORA^*b*^ (AD)*	chr15:60803740	NM_134261:c.505C>T;p.Q169X	het	*De novo*	
**065 F**	3.5 m	West syndrome	*SLC35A2 (AD)*	chr20:62070997	NM_001282651:c.550_552delTCC;p.S184del	het	*De novo*	Galactose trial: 6 months later more alert and interactive; no change in seizure frequency
071 F	46.5 m	Unclassified	*HNRNPH2^*b*^ (XLD)*	chrX:100667593	NM_01959:c.617G>A;p.R206Q	het	Not ♀, ♂ NA	
072 M	29.2 m	Unclassified	*TCF20^*b*^ (AD)*	chr22:42611134	NM_181492:c.178C>T;p.R60X	het	*De novo*	
073 F	12 m	EE	*YWHAG^*b*^ (AD)*	chr7:75959244	NM_012479:c.394C>T;p.R132C	het	*De novo*	
077 F	2.1 m	West, LGS	*CDKL5 (XLD)*	chrX:18622288	NM_003159:c.1245_1246delAG;p.E416VfsTer2	het	*De novo*	
093 F	21.4 m	EE	*SMARCA2^*a*^ (AD)*	chr9:2081857	NM_139045:c.2210C>A;p.S737Y	het	*De novo*	
**094 F**	7 m	Dravet	*SCN1A (AD)*	chr2:166915157	NM_001165963:c.305delT;p.F102SfsTer10	het	*De novo*	Changed medications: added clobazam and stiripentol
**100 F**	10.1 m	Unclassified	*ATP1A3 (AD)*	chr19:42471896	NM_152296:c.2839G>A;p.G947R	het	*De novo*	No change in management. Already on Flunarizine
106 F	3.7 m	EE	*STXBP1 (AD)*	chr9:130413885	NM_003165:c.41T>G;p.I14S	het	*De novo*	
113 F	7.5 m	West syndrome	*DYNC1H1 (AD)*	chr14:102469031	NM_001376:c.4700G>A;p.R1567Q	het	*De novo*	
**123 F**	11.8 m	EE	*GABRA1 (AD)*	chr5:161309644	NM_000806:c.604C>T;p.R214C	het	*De novo*	Based on functional studies, will be started on new medication (pending publication)
126 F	60 m	Unclassified	*RORA^*b*^ (AD)*	chr15:60803459	NM_134261:c.785dupG;p.E263RfsTer20	Het	*De novo*	
130 F	1.1 m	West syndrome	*DEPDC5 (AD)*	chr22:32239187	NM_001242896:c.2623delT;p.Y875Tfs46	het	*De novo*	
132 M	28.1 m	CSWS	*CNKSR2 (XLR)*	chrX:21609267	NM_014927:c.1785G>A;p.W595X	hemi	♀carrier	
137 F	24 m	Unclassified	*DYRK1A (AD)*	chr21:38865409	NM_001396:c.1042G>A;p.G348R	het	Not ♂, ♀ NA	
138 F	18 m	EE	*DCX (XLD)*	chrX:110653428	NM_178151:c.199G>A;p.G67R	het	*De novo*	
148 F	12 m	Unclassified	*DYRK1A (AD)*	chr21:38865409	NM_001396:c.763C>T;p.R255X	het	*De novo*	
152 F	7 m	EE	*NEXMIF^*b*^ (XLD)*	chrX:73962510	NM_001008537:c.1882C>T;p.R628X	het	unknown	
**158 F**	26.4 m	MAE/Febrile seizures plus	*SCN1A (AD)*	chr2:166848429	NM_001165963:c.298T>G.F100V	het	*De novo*	No change in management
160 F	27.5 m	EE	*MECP2 (XLD)*	chrX:153296777	NM_004992:c.502C>T;p.R168X	het	*De novo*	
168 F	41.5 m	EE	*MECP2 (XLD)*	chrX:153296857	NM_004992:c.422A>C;p.Y141S	het	*De novo*	
171 F	10 m	EE	*HNRNPU (AD)*	chr1:245025823	NM_031844:c.817C>T;p.Q273X	het	*De novo*	
**208 F**	10.8 m	Unclassified	*PCDH19 (XLD)*	chrX:99662976	NM_001184880:c.619_620delCGinsA;p.R207KfsTer5	het	*De novo*	Trial of steroids, but no response
**213 M**	1 day	Unclassified	*ATP1A3 (AD)*	chr19:42471896	NM_152296:c.2443G>A;p.E815K	het	*De novo*	Started Flunarizine with reduced hemiplegic episodes
**230 F**	3.5 m	West, LGS	*SCN8A (AD)*	chr12:52099304	NM_014191:c.1238C>A;p.A413D	het	*De novo*	Sodium channel blocker tried, not continued
**248 F**	37 m	Unclassified	*SCN1A (AD)*	chr2:166909410	NM_001165963:c.646A>T;p.R216X	het	*De novo*	Influenced choice of future treatment (stiripentol, CBD oil)

a *Variants identified by trio WES*.

b *Variants identified by WES reanalysis*.

*AAO, Age At Onset; AD, autosomal dominant; AR, autosomal recessive; CSWS, Epileptic Encephalopathy with continuous spike-and-wave during sleep; D, days; EE, unspecified Epileptic Encephalopathy; FS, Febrile seizure; IP, inheritance pattern; LGS, Lennox-Gastaut syndrome; MAE, Epilepsy with myoclonic-atonic seizures; M, months; NA, not available; SE, status epilepticus; SLFNE, Self-limited familial neonatal/infantile epilepsy; XL, X-linked; Zyg, zygosity; het; heterozygous; hom, homozygous; hemi, hemizygous*.

c *c.1029+1_1029+2delGTinsAA disrupts the canonical splice donor site of exon 12 and is predicted to abolish normal splicing*.

*Patients highlighted in bold are those for whom a genetic diagnosis had treatment implications*.

### Diagnostic Yield

A molecular diagnosis was established in 59/180 patients (33%) (Table 1). Pathogenic/likely pathogenic variants were identified in 41 genes. The majority of the diagnosed patients (41/59) had an autosomal-dominant disorder; of these 33 had a *de-novo* variant and remaining inherited from an affected parent and/or inheritance status unknown for one or more parent. Five patients had an autosomal-recessive disorder and the remaining 13 patients had an X-linked disorder (10 patients with X-linked dominant variants and 3 male patients with maternally inherited X-linked recessive disorders). In addition, a variant of uncertain significance (VUS) possibly explaining the clinical symptoms of the index patient was identified in 21 cases (12%) (Supplementary Table 3).

The diagnostic yield was higher in the Prospective (40%) than Retrospective group (30%). Patients in whom a diagnosis was made had earlier onset epilepsy (mean 13.2 vs. 21.9 months), and an epileptic encephalopathy was more common (Table 3). Of 82 patients with epileptic encephalopathy a definite or likely pathogenic variant was identified in 36 (44%) (Supplementary Table 4).

**Table 3:** Clinical features in patients with and without a genetic diagnosis.

**Genetic Diagnosis^**a**^**	**Mean AAO (range)**	**Males (%)**	**EE (%)**	**Treatment Resistant^**b**^ (%)**	**GDD/ID (%)**	**Autism (%)**	**MRI Abnormal (%)**
Definite or Likely (*n* = 59)	13.2 months (0.03–60)	39	61	80	85	25	37
No Diagnosis (*n* = 100)	21.9 months (0.03–60)	43	46	81	76	20	30

a *Individuals with variants of unknown significance or a possible genetic diagnosis are excluded (8)*.

b *Treatment resistant refers to failure to respond to 2 or more appropriate anti-seizure medications*.

*AAO, Age at onset; EE, Epileptic Encephalopathy; GDD/ID, Global Developmental Delay/Intellectual Disability*.

### Treatment Implications

A genetic disorder with specific therapeutic implications was diagnosed in 27 patients and management was influenced and/or altered in 23 (12 Prospective and 11 Retrospective). Clinical information, treatment changes, and impact are summarized (Table 2).

### Comparative Time to Diagnosis in First 50 Patients

The mean time to genetic diagnosis from study enrolment with genetic counseling to research validation of the variant was 38 days (20–70) for the Prospective group, 54 days (22–105) for the Retrospective group, and 47 days (20–105) overall. The mean time from epilepsy diagnosis to research validation of genetic diagnosis was 145 days (42–242) for the Prospective group and 2,882 days (429–7,686) or ~8 years for the Retrospective group.

### Cost Analysis in First 50 Patients

Point estimates and 95% confidence intervals based on bootstrapped standard errors (1,000 times with replacement) for each category of diagnostic test by cohorts were calculated (Table 4). All cost estimates use rates effective for the 2014–2015 fiscal year. The mean total cost related to the diagnosis of epilepsy was $4,524 (range $1,223–$7,852) for the Prospective cohort and $8,344 (range $3,319–$17,579) for the Retrospective cohort. Diagnostic imaging and electrophysiological tests comprise >60% of total epilepsy-related diagnostic costs. The mean for diagnostic imaging testing constituted $1,391 and $3,276, for Prospective and Retrospective cohorts, respectively. The mean for electrophysiological testing constituted $1,353 and $2,731, for Prospective and Retrospective cohorts, respectively. Our alternative scenario for diagnostic testing is MRI, EEG, chromosome microarray (CMA) and WES testing with Sanger sequencing validation, which amounts to $3,234 per patient (Supplementary Table 5). The difference in mean total cost related to the diagnosis of epilepsy for Prospective ($4,524) and Retrospective ($8,344) groups, exceeds the cost of our diagnostic alternative ($3,234). The potential average savings of targeted WES in the diagnostic workup constitute $1,290 per Prospective patient and $5,110 per Retrospective patient.

**Table 4:** Average diagnostic investigation cost per patient.

	**Mean**	**Bootstrap Std. Err**.	**95% CI**		**Range**	
					**Min**	**Max**
**COMBINED DIAGNOSTIC COST**						
Retrospective	$8,344.27	$556.97	$7,252.61	$9,435.90	$3,318.50	$17,578.8
Prospective	$4,524.07	$497.57	$3,548.85	$5,499.29	$1,223.18	$7,852
**LAB TESTS**						
Retrospective	$1,333.5	$83.71	$1,169.42	$1,497.56	$123.30	$3,129.91
Prospective	$1,151.32	$135.14	$886.43	$1,416.19	$209.75	$1,959.19
**GENETIC TESTS**						
Retrospective	$1,179.55	$98.80	$985.90	$1,373.20	0	$2,279.34
Prospective	$633.187	$144.27	$350.41	$915.96	0	$1,720
**DIAGNOSTIC IMAGING**						
Retrospective	$3,276.10	$214.10	$2,856.47	$3,695.74	$1,460	$6,836
Prospective	$1,391.38	$150.26	$1,096.86	$1,686	$630	$2,290
**ELECTROPHYSIOLOGICAL**						
Retrospective	$2,731.22	$376.26	$1,993.75	$3,468.70	0	$8,460.45
Prospective	$1,353.12	$315.64	$734.4	$1,971.78	$188.01	$3,572.19

## Discussion

High-throughput sequencing has been proven to be a great diagnostic tool in clinical practice in a variety of genetic conditions, and if used early in the diagnostic workup can lead to a reduction of costs associated with obtaining a molecular diagnosis (22). Gene panel sequencing is often favored over WES based on diagnostic yield, higher coverage and cost-savings (1). However, direct comparisons of diagnostic yield and/or costs of gene panel testing are limited. A comparative coverage analysis restricted to disease-causing variants identified through panels demonstrated that targeted WES detects ≥98.5% of those mutations (44), and targeted WES has been recently shown to have a higher diagnostic yield compared to gene panels (45). A major advantage of WES over panels is the ability to sequence the entire coding genome. Such comprehensive assessment can facilitate re-analysis for novel genes as they are implicated. In the course of this study several new epilepsy genes were identified by other research groups and published in the medical literature. We were able to go back to the original WES data and examine those genes in patients without a diagnosis. Re-analysis of WES data identified new diagnoses in eight patients (014, 044, 063, 071, 072, 073, 126, 152) and three additional diagnosis (013, 093, 144) were identified by trio WES, overall increasing the diagnostic rate from 27 to 33%. Given its static nature, panel sequencing is unable to include such contemporary targets. Newly discovered genes cannot be added to the test without re-design and validation, ultimately reducing the cost-effectiveness of gene panel testing.

The clinical utility of targeted WES with Sanger validation (limited ≤3 variants/exome) is supported by the identification of a definite or likely diagnosis in 59/180 (33%) patients and a possible diagnosis in an additional 21/180 (12%) (Table 1). A higher yield was found in the Prospective group with new-onset epilepsy and supports earlier testing, though the number of patients is small. However, this may also reflect increased severity of the disease, survival and potentially some bias in case referral. The Retrospective group had already undergone extensive clinical testing that was non-diagnostic. Nevertheless, our ability to still identify a genetic diagnosis supports the technology's superior resolution, while related data on phenotypes, management and outcomes may yet inform clinical practice.

The diagnostic yield in our study is comparable to previous findings (2–11). Most variants were *de-novo* and the genetic causes identified were heterogeneous. However, multiple variants were identified in several genes with the most common being *SCN1A*, followed by *KCNQ2* and *MECP2* (Table 2). In a comparable cohort, positive results were identified by WES in 112/293 (38.2%) epilepsy patients (3). We concur that the diagnostic yield is likely affected by the characteristics of the group studied, sample size, platform used (gene panel or WES) and the timing of the study, given ongoing gene discoveries in epilepsy. In our study, patients with a genetic diagnosis were younger and more likely to have an epileptic encephalopathy when compared to patients in which no genetic cause was found. Similar to a prior study (3), patients with an epileptic encephalopathies had a high rate of positive findings (44%).

Our results support the feasibility of targeted WES to rapidly provide clinically-confirmed genetic diagnoses in early-onset epilepsy. Time to Sanger sequencing validation from enrollment averaged 7 weeks which is similar to the 6–8 week turn-around-time quoted by most commercial testing labs. However, this estimate did not include the additional time required to obtain provincial government approval, on a case-by-case basis, to fund WES.

A timely genetic diagnosis is important when considering the potential for treatment impact and optimization of patient outcomes. Twenty-seven of the fifty nine patients with a genetic diagnosis (46%) had a disorder with specific treatment implications; for 23 patients an immediate change in medical management was made (Table 2). The number of genetic disorders identified to have specific treatments implications is likely to grow with ongoing advances in precision medicine.

In British Columbia, the average savings are estimated to be between $1,290 and $5,110 per patient, depending on whether they are new Prospective referrals or Retrospective. Of note, price estimates reflect academic and/or hospital costs rather than commercial costs which are 1–5X higher. The Canadian sequencing costs cited are comparable to previous reports but will decrease as even higher throughput sequencing technologies become accessible (15–18). Current healthcare cost estimates are also conservative as patients without a genetic diagnosis will undoubtedly require additional clinic visits and inpatient hospital stays, including epilepsy monitoring unit admissions related to finding the cause of their condition. Of note, a targeted WES approach did not lead to a substantial increase in referrals for incidental findings. Overall, our findings show targeted WES may provide an effective end to an otherwise invasive, time consuming and costly diagnostic odyssey, with societal and economic benefits. Our results also support WES implementation beyond early-onset epileptic encephalopathies as we have examined a larger and more diverse group of children (18).

### Limitations and Strengths

Our study has several limitations including small sample size although our diagnostic yield is comparable to previous studies. Incomplete coverage of the 620 genes analyzed was partially addressed as outlined in the methods. Proband-parent trio-based WES analyses were not used primarily for financial reasons. Analysis was restricted to 620 epilepsy genes, rather than the entire exome, to identify a genetic diagnosis as quickly as possible and to minimize secondary findings. Assessing relevance of secondary findings and proving pathogenicity of variants in novel candidate epilepsy genes is costly; thus, this approach was taken to maximize patient care and minimize cost. WES data from patients with initial negative results continues to be periodically reviewed for variants in newly described epilepsy genes. In subsequent WES trio analysis, a subset of families has helped identify novel genetic etiologies (41). All rare variants were considered however the Ion Proton™ sequencing platform tends to make mis-leading variant calling errors with small in/dels and homo-polymer stretches, with an excess of between 58 and 76% false positive calls (46). We have also identified instances of negative calls. Illumina sequencing platforms have better performance but assessing exon dosage from exome data is challenging at this sequencing depth. Panel sequencing, designed for more uniform coverage and greater depth, would be advantageous if the candidate gene was included or whole genome sequencing given the ability to detect all variants albeit for greater cost at lower coverage. Limited demographic information was provided for on the majority of patients and may be helpful in assessing variant frequencies in specific ethnic groups. Unfortunately, the ability to infer ancestry information from exome data is rather limited, in contrast to high-density SNP arrays, and not entirely reliable.

Although a significant number of genetic diagnoses had potential treatment implications, the long-term impact on clinical outcome following genetically-informed therapeutic interventions is unknown. Early diagnosis and early intervention are important, but advances in precision medicine are also required.

The methods employed for cost analysis cannot replace a prospective randomized controlled trial (RCT) and may not have accurately assessed or included all healthcare costs related to an epilepsy diagnosis. However, an RCT assessing the effect of WES testing on healthcare costs is not yet a practical consideration. Our estimates are not a perfect or a complete description of the current diagnostic work-up, as test records are scattered across different electronic health records systems and paper charts. Data collation within an accessible unified health electronic record would help identify where additional savings are possible. In this study, indirect costs, and the psychosocial impact on the child and family were not measured.

## Conclusion/Summary

Targeted WES with limited Sanger sequencing validation is a rapid and minimally invasive test with potential to save costs within the Canadian healthcare system. An early genetic diagnosis may improve a patient's clinical outcome and quality of life. Further research on larger cohorts is warranted to inform diagnosis, clinical outcome and precision medicine. Acknowledging the limitations of our study, targeted WES with Sanger sequencing validation substantially improves current practice and is recommended as the dominant diagnostic strategy in early onset epilepsy. Minimally, as high-throughput sequencing costs continue to fall, trio-based whole exome sequencing reporting (and potentially re-reporting if negative), averting the need for Sanger validation for *de novo* variants, should be implemented as a first-line test strategy in British Columbia.

## Ethics Statement

This study was carried out in accordance with the recommendations of BC Children's Hospital and University of British Columbia Ethics Board with written informed consent from all subjects. All subjects gave written informed consent in accordance with the Declaration of Helsinki. The protocol was approved by the BC Children's Hospital and University of British Columbia Ethics Board (protocol number H14-01531).

## Author Contributions

MD contributed to study conception and design, data analysis and interpretation, review of patients clinically, obtaining funding, drafting the manuscript. IG contributed to genetic data acquisition, analysis and interpretation, revised the manuscript. CD contributed to data acquisition, analysis and interpretation, and revised the manuscript. MM contributed to genetic data acquisition and to data analysis. CyB contributed to clinical assessments, data acquisition and interpretation, revised the manuscript. DE performed bioinformatics analysis. SB designed REDCap database, contributed to data acquisition, analysis and interpretation, revised the manuscript. ET contributed to data acquisition and organization. LH, AD, AM, KS, EL-R, and BB contributed to clinical assessments and data acquisition/interpretation. GH and RS performed biochemical clinical assessments, contributed to data interpretation. CvK contributed to study design and variant interpretation. ES assisted preparation of execution of study. PE performed cytogenetic assessment. SA performed genetic counseling, contributed to data acquisition and literature review. MV contributed to genetic counseling, review of results and literature, and revising manuscript. TN contributed to data analysis and interpretation, performed clinical variant interpretation, revised the manuscript, negotiated funding for variant clinical validation. CoB performed economics data acquisition, analysis and interpretation; drafted economics section of manuscript. MC performed electroclinical phenotyping, revised the manuscript, obtained funding. MF contributed to study conception and design, data analysis and interpretation, obtained funding, and revised the manuscript.

### Conflict of Interest Statement

MD has received research support from the Rare Disease Foundation and the Alva Foundation. TN has received research support from the BCCH Foundation and Genome BC. MC has received research grants and/or speakers honoraria from UCB, Novartis, Biocodex, Eisai, and Sage Therapeutics. All honoraria are donated to the Epilepsy Research and Development Fund. She has also received research grants from CIHR (Canadian Institute for Health Research) and The Alva Foundation. She is Co-Chair of the Canadian Pediatric Epilepsy Network. MF was a founding partner Neurocode Labs Inc., that currently provides commercial exome sequencing, but has since relinquished any ownership stake in that company. The remaining authors declare that the research was conducted in the absence of any commercial or financial relationships that could be construed as a potential conflict of interest.

## Appendix

**List of Relevant Tests:**

**Laboratory Services/Tests:**

- Bloodspot acylcarnitines
- Plasma amino acids
- Plasma total homocysteine
- Serum ceruloplasmin
- Serum copper
- Ammonia Serum
- Lactate Serum/plasma
- Lactate whole blood
- Acylcarnitine Serum
- Homocysteine total from Plasma
- Plasma amino-acids
- Serum copper
- Serum coeruloplasmin
- Plasma very long chain fatty acids
- Plasma cholesterol
- Urine creatine metabolites
- Urine glycosaminoglycans
- Urine oligosaccharides
- Urine organic acids
- Urine purines and pyrimidines
- Amino acids-urine
- Urine creatine metabolites
- Urine mucopolysaccharides
- CSF Protein
- CSF Protein
- CSF Glucose
- CSF Cell
- CSF Lactate
- CSF Amino-Acids
- CSF Neurotransmitters
- Plasma Vit B12

**Genetic Tests:**

- Chromosome microarray
- Fluorescence *in situ* Hybridization
- Single Gene testing
- Gene Panel/HTS testing
- Mitochondrial DNA analysis

**Diagnostic Imaging Tests:**

- MRI
- CT
- PET
- PET/CT
- Ultrasound **Electrophysiological Tests:**
- EEG
- EMG

**Biopsies:**

- Skin biopsy
- Muscle biopsy
