# Supplementary material for: Diagnostic Yield and Treatment Impact of Targeted Exome Sequencing in Early-Onset Epilepsy
Source: Front Neurol. 2019 May 21;10:434. doi: 10.3389/fneur.2019.00434 (PMC6536592; doi:10.3389/fneur.2019.00434)
Supplement: Supplementary file 3 [file Table_3.docx]

**Supplementary Table 3: Variants of Uncertain Significance.**

| **ID** | **AAO** | **Epilepsy** | **Gene**  **(IP)** | **Transcript RefSeq ID** | **NT** **Change** | **AA** **Change (CADD)** | **Zyg** | **Inheritance** |
| --- | --- | --- | --- | --- | --- | --- | --- | --- |
| 007R | 38.3 m | MAE | *NDST1*  *(AR)* | NM_001543 | c.1766A>G | p.K589R (23.2) | comp het | ♀carrier |
|  |  |  |  |  | c.2218G>A | p.A740T (17.5) |  | ♂carrier |
| 016R | 24.4 m | EE | *ARHGEF9*  *(XLR)* | NM_015185 | c.1315G>T | p.E439X (26.2) | het | De novo |
| 019R | 30 m | Unclassified | *CHD2*  *(AD)* | NM_001271 | c.1843G>C | p.G615R (28) | het | NA |
| 025R | 5.1 m | Unclassified | *HUWE1*  *(XLR)* | NM_031407 | c.9410A>C | p.N3137T (17.5) | hemi | ♀carrier |
| 030R | 37.8 m | CAE | *DIP2B ^a^*  *(AD)* | NM_173602 | c. 182C>G | p.S61X (37) | het | De novo |
| 052R | 2 m | Unclassified | *WDFY3 ^b^*  *(AD)* | NM_014991 | c.7605+3A>G | NA^a^ | het | De-novo |
| 038R | 13 m | MTLE with HS | *SCN1B*  *(AD)* | NM_001037 | c.206A>C | p.K69T (20.9) | het | ♀carrier |
| 057R | 0.8 m | Unclassified | *KCNQ2*  *(AD)* | NM_172107 | c.2555C>T | p.P852L (23.5) | het | ♂carrier |
| 066R | 12 m | Unclassified | DSCAM *^b^* | NM_001271534 | c.2573A>G | p.E858G (21.2) | het | De-novo |
| 076R | 36 m | EE | GNAI1 *^b^* | NM_002069 | c.614_615delGA | p.R205IfsTer2 | het | ♂carrier |
| 091R | 4.3 m | Unclassified | *SETD5 ^b^*  *(AD)* | NM_001080517 | c.1052G>T | p.R351I (29.6) | het | De-novo |
| 102R | 15.5 m | Unclassified | ARSH *^b^* | NM_001011719 | c.655C>T | p.R219X (35) | hemi | ♀carrier |
| 107R | 60 m | CSWS | EP400 *^a^* | NM_015409 | c.8558G>A | p.R2853Q (35) | comp het | ♀carrier |
|  |  |  |  |  | c.6097C>T | p.P2033S (21.4) |  | ♂carrier |
| 131P | 5.1 m | Unclassified | *GABRB3 (AD)* | NM_000814 | c.881G>A | p.R294Q (35) | het | Not ♀, ♂ NA |
| 145R | 18 m | Unclassified | TRRAP *^b^* | NM_001244580 | c.816C>A | p.Y272X (37) | het | ♂carrier |
| 147R | 7.9 m | EE | *TAF1 ^b^*  *(XLR)* | NM_004606 | c. 2771A>G | p.Y924C (28.5) | hemi | ♀carrier |
| 149P | 6.9 m | Unclassified | *KCNQ2*  *(AD)* | NM_172107 | c. 1966G>A | p.E656K (24.5) | het | ♂carrier |
| 150R | 3 m | EE | *CACNA1A*  *(AD)* | NM_001127221 | c.1242G>T | p.R414S (23.6) | het | Not ♀, ♂ NA |
| 196P | 4.2 m | Unclassified | *ARHGAP32* *^a^* | NM_001142685 | c.1916T>C | p.I639T (28.1) | het | De novo |
| 202P | 5 m | Unclassified | *SIN3A* *^b^*  *(AD)* | NM_001145358 | c.3418C>T | p.R1140X (42) | het | De-novo |
| 207R | 22 m | Unclassified | *CACNA1E ^b^*  *(AD)* | NM_000721 | c.4763G>A | p.R1588H (34) | het | unknown |

^a^ Variants identified by trio WES; ^b^ Variants identified by WES reanalysis; m = months; AA = amino acid change; AD = autosomal dominant; AR = autosomal recessive; CAE = Childhood Absence Epilepsy; CSWS = Epileptic encephalopathy with continuous spike-and-wave during sleep; EE = unspecified Epileptic Encephalopathy; IP = inheritance pattern; LGS = Lennox-Gastaut Syndrome; MAE = Epilepsy with myoclonic-atonic seizures; MTLE with HS = mesial temporal lobe epilepsy with hippocampal sclerosis; CADD = Combined Annotation Dependent Depletion score (cadd.gs.washington.edu) NA = not available; NT = nucleotide change; P = prospective; R = retrospective; VUS = Variants of Uncertain Significance; XL = X-linked.
