# Supplementary material for: Diagnostic Yield and Treatment Impact of Targeted Exome Sequencing in Early-Onset Epilepsy
Source: Front Neurol. 2019 May 21;10:434. doi: 10.3389/fneur.2019.00434 (PMC6536592; doi:10.3389/fneur.2019.00434)
Supplement: Supplementary file 4 [file Table_4.docx]

**Supplementary Table 4: Diagnostic Yield in 82 patients with an Epileptic Encephalopathy**

| **Syndrome** | **Number of Cases** | **Definite/Likely Diagnosis (%)**  **Genes** |
| --- | --- | --- |
| Dravet | 6 | 5 (83%)  ***SCN1A*** |
| West Syndrome | 23 | 7 (30%)  ***ALG13, PAF2H1B1, SLC35A2, DYNC1H1, ADSL, DEPDC5, ARX*** |
| West S./LGS | 5 | 2 (40%)  ***CDKL5, SCN8A*** |
| LGS | 6 | 1 (17%)  ***GABRB3*** |
| Ohtahara S./West | 1 | 1(100)  ***STXBP1*** |
| MAE | 4 | 1(25%)  ***SCN1A*** |
| CSWS | 4 | 1(25%)  ***CNKSR2*** |
| LKS | 2 | 0 |
| EE unclassified | 31 | 18 (58%)  ***ARHGEF9, KCNQ5, PIGA, POLG, PNPT1, PIGA, KCNQ2 (x2), STXBP1, SLC1A2^29^, YWHAG, SMARCA2, GABRA1, DCX, NEXMIF, MECP2 (x3), HNRNPU, SCN1A*** |

CSWS=Epileptic Encephalopathy with continuous spike-and-wave during sleep; EE= Epileptic Encephalopathy; LKS= Landau-Kleffner syndrome; LGS=Lennox-Gastaut syndrome; MAE= Epilepsy with myoclonic-atonic seizures.
