# Supplementary material for: Diagnostic Yield and Treatment Impact of Targeted Exome Sequencing in Early-Onset Epilepsy
Source: Front Neurol. 2019 May 21;10:434. doi: 10.3389/fneur.2019.00434 (PMC6536592; doi:10.3389/fneur.2019.00434)
Supplement: Supplementary file 5 [file Table_5.docx]

**Supplementary Table 5: Costs**

| **Test** | **Cost^a^** |
| --- | --- |
| MRI | $686 |
| EEG | $188 |
| CMA | $860 |
| WES & Sanger sequencing | $1500 |

^a^ Price estimates reflect academic and/or hospital costs rather than commercial costs. CMA: chromosome microarray, WES: Whole-exome sequencing
